# Comparative study on the clinical efficacy of small plate assisted anatomic plate and traditional double plate in the treatment of Rüedi and Allgöwer II - III pilon fracture

**DOI:** 10.1186/s12893-023-02251-9

**Published:** 2023-11-18

**Authors:** Wenbin Ding, Jianing Xu, Ya Zhu, Shensheng Nian, Yifeng Lu, Miaomiao Zheng, Xiang Zhu, Zimin Wang, Fang Ji, Hao Tang

**Affiliations:** 1 Department of Orthopedics, Changhai Hospital, Naval Military Medical University, 168 Changhai Road, Shanghai, China; 2 Department of Orthopedics, The Second People’s Hospital of Huai’an, The Affiliated Huai’an Hospital of Xuzhou Medical University, Huai’an, 223003 Jiangsu Province China; 3 Department of Orthopedics, The Ninth People’s Hospital, Shanghai Jiaotong University, Shanghai, China

**Keywords:** Pilon fracture, Small plate, Rüedi and Allgöwer II - III

## Abstract

**Objective:**

The aim of this study was to investigate the clinical efficacy of small plate assisted anatomical plate and traditional double plate in the treatment of Rüedi and Allgöwer II - III pilon fracture.

**Methods and materials:**

The data of 68 patients with pilon fracture admitted to Hospital from June 2017 to June 2020 were retrospectively analyzed. Study group and control group were divided according to different operation methods, with 34 cases in each group. There were 28 cases of Rüedi and Allgöwer II type and 40 cases of Rüedi and Allgöwer III type. Perioperative period data, Ankle joint function score, visual analog scale (VAS) scores and the incidence of incision complications were analyzed between these two groups.

**Results:**

There were no significant differences in full load time, fracture healing time between these two groups (*P* > 0.05). The operation time, intraoperative blood loss, length of hospital stay, Ankle joint function score and postoperative incision complication rate in observation group were lower than those in control group (*P* < 0.05).

**Conclusion:**

Small plate assisted anatomic plate is comparable to traditional double plate in the treatment of pilon fracture in terms of complete loading time, fracture healing time, but the former can shorten the operation time, reduce intraoperative blood loss and effectively reduce the incidence of postoperative complications.

## Introduction

 Pilon fracture is a fracture of the lower tibia involving the lower articular surface of the tibia, which is characterized by a typical comminuted distal tibia fracture of varying degrees, involvement of the articular surface, primary damage to the articular cartilage, and a poor prognosis due to permanent articular surface irregularity [1, 2]. Therefore, the treatment of Pilon fracture has always been a difficult and hot spot in orthopedic trauma. Rüedi and Allgöwer were among the first trauma surgeons to study pilon fracture. Furthermore, they developed a classification system that identified pilon fractures into three distinct categories, based on the degree of comminution and articular surface displacement. However, Rüedi - Allgöwer type II ~ III fracture is very difficult to be treated clinically [3, 4]. At present, surgical treatment is still the preferred treatment for pilon fracture, but good intraoperative exposure of the fracture and the articular surface, good reduction of the fracture, reasonable and effective fixation, intraoperative soft tissue protection and postoperative joint function recovery are the key and difficult points of treatment [5]. The most significant characteristics are comminuted and unstable fractures accompanied by the destruction of the articular surface, less soft tissue around the fracture and more serious injuries, and poor blood flow [6]. Therefore, pilon fracture is one of the most difficult fractures to manage clinically and deserves to be improved.

Thus, the purpose of this study was to investigate the clinical efficacy of small plate assisted anatomical plate and traditional double plate in the treatment of Rüedi and Allgöwer II - III pilon fracture. The baseline date, perioperative period data, Ankle joint function score, visual analogue scale (VAS) scores and the incidence of incision complications were analyzed between these two groups.

## Methods and materials

### Patients

#### Inclusion and exclusion criteria

Inclusion criteria: (1) Pilon fracture. (2) Consistent with surgical indications. (3) The soft tissue condition was good (For open fractures, we need to determine the soft tissue surrounding the fracture site and define good soft tissue condition by confirming as Gustillo-Andersen Type I or Type II fracture.).

Exclusion criteria: (1) Patients with hematological diseases. (2) Patients with infectious disease. (3) Patient with congenital developmental malformation of the ankle joint. (4) Patients with talus or calcaneal fractures. (5) Gustillo-Andersen Type III fracture.

### Preoperative Preparation

All the patients underwent elective surgery, which included manual reduction, temporary fixation with plaster support or calcaneal traction at the early stage to maintain a neutral ankle position and restore the length of the affected limb as much as possible. Then, patients were instructed to elevate the affected limb, apply local ice, and take oral drugs to reduce swelling. After 7–14 days, surgery was performed when local soft tissue swelling subsided and skin wrinkles developed. All patients received anteroposterior and lateral ankle radiographs and three-dimensional ankle CT scan before surgery [7, 8]. There was no significant difference in intraoperative reduction or postoperative score between calcaneal traction or plaster fixation.

### Surgery procedures

#### Control group

**Fig. 1:**
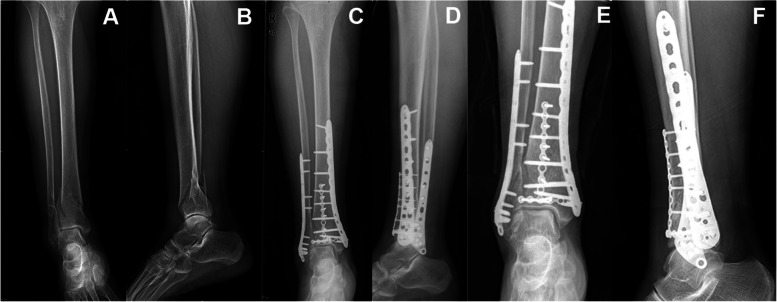
A 41-year-old male with pilon fracture of his left leg from a height fall, Rüedi and Allgöwer II type. **A**, **B** Preoperative anteroposterior and lateral radiographs. **C**, **D** Anteroposterior and lateral view of postoperative X-ray. **E**, **F** Anteroposterior and lateral X-ray 15 months after surgery

Patients were given epidural anesthesia. Patients with pilon fracture complicated with fibular fracture were first treated with open reduction and internal fixation of fibular fracture to restore the fibular force line. Then, according to the fracture area and fracture classification, anterior median incision or anterolateral incision was used for exposure, and two plates (distal tibial anatomic plate or reconstruction locking plate) were used for fixation (Fig. 1).

### Study group

**Fig. 2:**
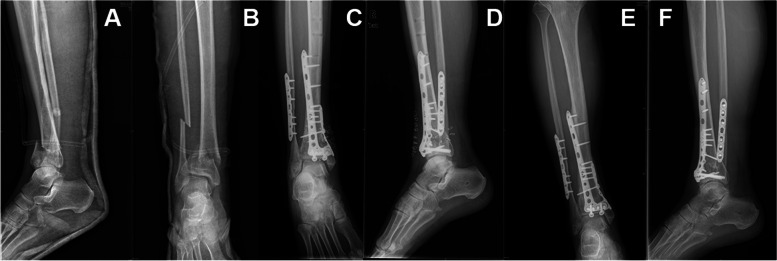
A 55-year-old female with pilon fracture of his left leg from a height fall, Rüedi and Allgöwer II type. **A**, **B** Preoperative anteroposterior and lateral radiographs. **C**, **D** Anteroposterior and lateral view of postoperative X-ray. **E**, **F** Anteroposterior and lateral X-ray 15 months after surgery

Patients were given epidural anesthesia. Patients with pilon fracture complicated with fibular fracture were first treated with open reduction and internal fixation of fibular fracture to restore the fibular force line. Then, according to the fracture area and fracture classification, medial or lateral anterior incision was used to expose the anterior lip of the tibia, medial malleolus and anterolateral Chaput bone mass. If necessary, the tibial talus joint was opened to expose the articular surface of the distal tibia to reduce the articular surface compression bone mass. If there was bone defect, the iliac bone graft was taken from the body. The incision distance was 3.5 ~ 8 cm, and the incision length was 3 ~ 8 cm. After reduction of the articular surface, the posterior column was fixed with a small steel plate (A.L.P.S. Hand Fracture System, ZIMMER BIOMET), and then the anterior medial column of Pilon fracture was fixed with an L-shaped anatomic plate to examine the stability of the ankle. If the stability was poor, the medial stability of the fracture was enhanced by small medial plate support. A negative pressure drainage tube was routinely placed inside the wound and the wound was sutured layer by layer (Fig. 2).

### Postoperative management

Routine prevention of infection, detumescence and thrombosis within 48 h after operation. Postoperative drainage tube was routinely removed within 48 h. No plaster or brace was used for postoperative fixation, and the affected limb was raised after surgery. On the first day after surgery, under the guidance of the rehabilitation physician, functional exercise was standardized and active flexion and extension of the hip, knee and ankle joint functional exercise was started. 6–8 weeks after the operation, weight-bearing exercise was determined gradually according to the fracture healing of X-ray film.

### Perioperative assessment

Operation time, intraoperative blood loss, hospital stay, full weight-bearing time, and fracture healing time were assessed.

### Postoperative evaluation

Burwell-Charnley imaging evaluation criteria were used to evaluate the quality of fracture reduction after surgery: excellent: no lateral or angular displacement of the internal and external malleolus, longitudinal separation and insertion < 1 mm, anteroposterior malleolus displacement < 2 mm, no talus displacement; Good: there was no lateral or angular displacement of the lateral malleolus, anteroposterior displacement of the lateral malleolus was 2 ~ 5 mm, anteroposterior displacement of the posterior malleolus was 2 ~ 5 mm, no talus displacement; Poor: any lateral lateral lateral lateral malleolus displacement, lateral malleolus anteroposterior displacement > 5 mm, or posterior malleolus displacement > 5 mm, with talus displacement.

Efficacy was evaluated according to the American Association of Foot and Ankle Surgery (AOFAS) ankle and hindfoot function score, including pain (40 points), function (50 points), and alignment (10 points): Excellent ≥ 90 points, good 75–89 points, fair 50–74 points, poor < 50 points.

Visual analogue scale (VAS) was used to evaluate the pain degree of the patient. According to the pain degree of patients, the full score was 10, 0 was recorded for painless pain, and 10 was recorded for severe pain. The higher the score was, the higher the pain degree of patients.

Postoperative complications, including skin necrosis, wound infection, redisplacement of fracture and traumatic arthritis, were recorded in both groups.

### Statistical analysis

We used SPSS 20.0 software to analyze data. Data were expressed as mean ± standard deviation. Paired sample t test was used for intra-group comparison and independent sample t test was used for inter-group comparison. *p* < 0.05 was considered statistically significant.

## Results

### Patient characteristics

Both thirty-four patients were included in this study, the average age was 42.74 ± 4.81 years old in the study group and 41.50 ± 4.66 years old in the control group. 19 patients were female in the study group and 18 patients were female in the control group. The main cause of injury was high falling injury in the both groups. All pilon fractures were classified by Rüedi and Allgöwer classification system, there were 22 patients with Rüedi and Allgöwer II pilon fractures and 12 patients with Rüedi and Allgöwer III pilon fractures in the study group. In the control group, there were 20 patients with Rüedi and Allgöwer II pilon fractures and 14 patients with Rüedi and Allgöwer III pilon fractures. v The baseline date of both groups was shown in Table 1, which demonstrated that patients in the distribution of each characteristic were similar in the two groups.

**Table 1:** The baseline date of the study group and the control group

Characteristics	Study group	Control group	*P*
Age (mean, years)	42.74 ± 4.81	41.50 ± 4,66	0.286
Gender			0.808
Male	15	16	
Female	19	18	
Cause of injury			0.870
Traffic injury	12	14	
High falling injury	17	15	
Sprain	5	5	
Rüedi and Allgöwer			0.816
Type II	22	20	
Type III	12	14	
Tscherne-Gotzen			0.808
Grade I	15	16	
Grade II	19	18	
Calcaneal traction	14	12	0.816

### Clinical data analysis

Operation time was 124.9 ± 9.99 min in the study group and 135.2 ± 3.53 min in the control group (*P* < 0.001, Table 2). Intraoperative blood loss was 116.3 ± 7.07 ml in the study group and 125.7 ± 1.41 ml in the control group (*P* < 0.001). Hospital stays was 19.00 ± 2.83 days in the study group and 21.59 ± 3.54 days in the control group (*P* < 0.005). Full Load was 16.21 ± 2.43 weeks in the study group and 16.33 ± 2.54 in the control group (*P* = 0.823). Fracture healing time was 15.41 ± 1.62 weeks in the study group and 15.59 ± 1.71 in the control group. Furthermore, we accessed ankle joint function and VAS score (Table 3). The ankle joint function score was 88.126 ± 6.04 in the study group and 86.01 ± 6.03 in the control group (*P* < 0.001). The VAS score was 6.41 ± 1.01 in the study group and 5.01 ± 1.13 in the control group (< 0.001). Moreover, Complication rates was 5.88% (2/34) in the study group and 23.5% (8/34) in the control group (*P* = 0.044) (Table 4).

**Table 2:** Perioperative period data of study group and control group

Clinical data	Study group	Control group	*P*
Operation time/ min	124.9 ± 9.99	135.2 ± 3.53	< 0.001
Intraoperative blood loss	116.3 ± 7.07	125.7 ± 1.41	< 0.001
hospital stays	19.00 ± 2.83	21.59 ± 3.54	< 0.005
Full load/ weeks	16.21 ± 2.43	16.33 ± 2.54	0.823
Fracture union time/ weeks	15.41 ± 1.62	15.59 ± 1.71	0.618

**Table 3:** Ankle joint function score of study group and control group

Characteristics	Study group	Control group	*P*
VAS score	6.41 ± 1.01	5.01 ± 1.13	< 0.001
Ankle joint function score	88.126 ± 6.04	86.01 ± 6.03	< 0.001

**Table 4:** Complication occurrence of study group and control group

	Study group	Control group	*P*
Skin necrosis	0	2	
Infection	0	1	
Fracture redisplacement	1	2	
Traumatic arthritis	1	3	
Total	2 (5.88%)	8 (23/5%)	0.044

## Discussion

In this study, we compared the clinical efficacy of small plate assisted anatomic plate and traditional double plate in the treatment of Rüedi and Allgöwer II - III pilon fracture. We found that there was no significant difference in full load time and fracture union time between these two groups. Operation time, intraoperative blood loss, hospital stays were significantly less than those of control group. Furthermore, VAS scores and ankle joint function score were better than those of control group. Moreover, The postoperative complication rate of study group was significantly lower than that of control group. The results revealed that small plate assisted anatomic plate is comparable to traditional double plate in the treatment of pilon fracture in terms of complete loading time, fracture healing time, but the former can shorten the operation time, reduce intraoperative blood loss and effectively reduce the incidence of postoperative complications.

The key to the treatment of pilon fracture are the operation time, reduction of fracture, protection of soft tissue and postoperative management. At present, there are many surgical fixation methods for pilon fractures, including clover plate, anatomic plate, locking compression plate internal fixation [9], minimally invasive percutaneous plate internal fixation [10], limited internal fixation plus external fixation [11] and so on. Since pilon fractures are usually high-energy injuries and often accompanied by serious soft tissue injuries, traditional incisions are long and damaged, and the larger steel plate is placed directly below the incision, which is easy to cause excessive skin tension at the edge of the incision and difficult closure, leading to skin necrosis and incision infection, and thus exposing the steel plate. It has been reported in the literature that the incidence of incision dehiscence and infection through traditional anterior medial approach can be as high as 55% [12]. The mechanical properties of classical clover plate and distal tibial anatomical plate are not good after fixation, and cannot achieve stable, effective and ideal internal fixation. MIPO technique [13] has little soft tissue damage and relatively low wound complications, but it is difficult to reduce high-energy pilon fractures with metaphyseal comminution and articular surface compression, and intraoperative bone grafting is difficult, which affects postoperative ankle function. External fixation scaffolds combined with limited internal fixation can be used to maintain bone length and line of force and reduce the incidence of complications such as infection. However, for type III pilon fractures with severe comminution, the reduction quality is difficult to guarantee, and it is easy to be redisplaced, which leads to long fracture healing time and high incidence of malunion and non-union.

In this study, part of type II and all type III fractures were multi-column fractures involving medial malleolus, anterolateral and medial tibia (Chaput bone block), distal fibula and posterior tibia (WagStaff bone block), and posterior malleolus triangle bone block (Volkmann bone block). A single anterior incision could not take into account all bone blocks. Therefore, when multiple incisions are used, the advantage of smaller plates used in combination can be realized if multiple conventional large plates are still used, which can have a greater impact on the soft tissue. Corresponding incisions were selected for different bone blocks to facilitate the exposure of fracture blocks and facilitate fracture reduction. Due to the use of small plates, the incision is short, 3 to 8 cm in length, and for some patients with longer fracture lines, a proximal auxiliary incision can be added. The incisions should not be separated as far as possible, and the periosteum should be limited to preserve the connection between the skin and subcutaneous and myofascial layers, which has little damage to the blood supply of the flap and is conducive to wound healing and wound related complications. The incision spacing can also be narrowed. In this group, the incision spacing was as short as 3.5 cm without flap necrosis. Some scholars have also reported that 7 cm cannot be used as an absolute indicator as long as the vascular distribution area is carefully considered before surgery to avoid damage to important vascular areas and gentle operation during surgery [12].

The treatment of Pilon fracture requires the rehabilitation of the articular surface and the articular surface of the distal tibia. L-shaped anatomical plate was used to fix the anterior medial column of Pilon fracture, which was used as the main plate to fix the anterior medial column, and a small plate was used to assist in fixing the posterior and medial columns. The small auxiliary steel plate we used has good compliance and easy shaping. After being pressurized by screws, it can fit completely with the bone surface and increase the friction force. Not only that, the volume of small plate is small, can avoid more peel, soft tissue coverage is easy, will not increase the skin tension, conducive to wound healing. It has been reported in the literature that for pilon fractures, ankle movement without weight bearing is required in the early postoperative period and weight bearing in the ground later, regardless of the fixation method. From another aspect also shows that the existing fixing method cannot achieve the effect of strong fixing. The clinical application results show that the small auxiliary plate can achieve stable fixation, effectively prevent the loss of reduction, and allow the ankle joint non-weight-bearing functional exercise in the early stage. Although this study has certain advantages in incision exposure and reduction of incision complications, due to insufficient sample size, the indications need to be further studied and improved by large-scale case and long-term follow-up data. The mechanical strength of pilon fractures assisted by small plates can be further tested by biomechanics. The internal fixation materials for distal tibial fractures can also be miniaturized and individualized, and the effects of different distal fracture blocks can be fixed in a targeted way, so as to achieve the purpose of effective and minimally invasive.

## Conclusion

Small plate assisted internal fixation in the treatment of Pilon fracture has the advantages of light soft tissue injury, small trauma and relatively reliable fixation, which can avoid soft tissue complications to the maximum extent and improve the fracture healing rate.

## Data Availability

All data generated or analysed during this study are included in this published article.
